# The Children's Hospital. Great Ormend Street

**Published:** 1893-03-11

**Authors:** 


### The Institutional Workshop.

### WITHIN THE HOSPITALS.

### THE CHILDREN'S HOSPITAL, GREAT ORMOND STREET.

(By Our Special Commissioner.)

The Right Honourable H. H. Fowler has wisely determined to make the workhouses more accessible to the friends of the inmates and also to the public than heretofore. It is our duty, in the course of each year, to inspect a large number of public institutions, and we know from personal observation that the most difficult place to enter for such a purpose is, as a rule, the workhouse and the poor law infirmary. With some notable exceptions, visitors of all kinds are discouraged, and a stranger who presents himself without credentials would certainly not receive facilities for making a careful inspection of the majority of British institutions under the poor law to-day. Of course, the results of this system of exclusion are anything but beneficial to the inmates, to the staff who are responsible for the management, and to the public interests involved. We could therefore wish that Mr. Fowler, as President of the Local Government Board, would issue an order which should ensure that any ratepayer who presented himself or herself at a reasonable hour should have free access to all poor law infirmaries, workhouses, and especially to the sick wards in workhouses. We have reason to believe, and the Local Government Board must have evidence to support the view, that there are a considerable number of sick and infirm wards connected with workhouses in the United Kingdom at the present time, which foster abuses and evils that would cause a tremendous outcry were they to be brought to the public notice in the newspapers. We commend these facts and observations to those members of the working classes and others who hold to the mistaken idea that all medical institutions should be supported out of the rates. The Metropolitan Asylums Board hospitals suffer in efficiency to a serious extent, owing to the absence of publicity which, in this case, is almost unavoidable. They are injured too by the fact that the managers, or certain of the committees of the managers, are opposed to the institution of a proper system of nursing which will secure the employment in all fever hospitals of trained nurses, and trained nurses only. We are led to make these remarks owing to a visit which we recently paid to the Hospital for Sick Children in Great Ormond Street. Arrived there without notice of any kind, we had simply to state the object of our visit to be conducted over every part of the institution without delay or difficulty. There is no voluntary hospital in the country where visitors are not welcomed and afforded the fullest facilities for seeing the whole establishment. No doubt to this fact is due much of the efficiency of the voluntary hospitals—an efficiency which far exceeds that to be met with in any other class of institution either in this country or elsewhere. It is, therefore, gratifying to be able to report that after making a careful inspection of every part of the Hospital for Sick Children, we were impressed with the feeling that the whole administration reflects great credit upon the committee and its officers, and that, so far as the patients at any rate are concerned, everything is done to secure the rapid recovery of the little sufferers. The accommodation for the staff, though fairly satisfactory, will not compare, however, with that provided for the nurses by the Edinburgh and Glasgow hospitals. This is a point which will no doubt receive attention at the hands of the committee, now that they have completed the new buildings which form such a splendid addition to this most popular hospital.

A Children's Hospital as an Educational Agent

We do not think that the public at present appreciate the educational power for good possessed by a wisely administered children's hospital. To its hospitable doors come the children of the slums of great cities, who, for the most part, have been sadly neglected, and are without cleanly habits, or, indeed, any fixed habits at all. Once passed by the doctors for in-patient treatment, a child is admitted to the receiving-room, and is there bathed and thoroughly cleansed and fumigated before being admitted to the wards. Its clothes are then disinfected, and so the child and its parents receive in many cases their first lesson in the doctrine that cleanliness is next to godliness. Once in the wards the discipline and teaching are continuously maintained during the whole time of the child's treatment, which lasts, on an average, for at least one month. At the end of that time the little patient is usually sent on to the convalescent branch for another month's careful treatment and training. Thus, for two months on an average, the child receives instruction which must have a more or less permanent effect upon its personal habits during the rest of its life. Every child in the wards is washed from head to foot once a day; it is taught to be well-behaved, well-conducted, and well-disciplined. It learns intuitively the comforts to be derived from cleanly surroundings and personal ablutions, and so is made to attach importance to these things, which before had probably formed no part of its education. Of course, the more careful the training the greater will be the cost per bed, but, having regard to the education given to the children, we are of opinion that good and permanent value is received for the increased expenditure. We take it that the committee of the Hospital for Sick Children are abundantly justified in providing three probationers, one staff nurse, and one sister for day duty alone in each of the large wards with 24 cots. Such a staff enables the work to be thoroughly well done, and secures a smartness and efficiency which must be absent where the convalescent children are largely left to their own resources. For these reasons we hope that the committees of children's hospitals throughout the country will carefully consider the influence for good which these institutions have the power of exercising on the rising generation to the fullest extent. No one can make a careful inspection of the Great Ormond Street hospital without being struck by the importance of the point we have dwelt upon.

Wards, New and Old.

The new wards, containing 24 cots, are magnificent. Magnificent, that is, in appearance, and magnificently kept. Not only the children, but the ward floors, walls and appurtenances, and also the cupboards, dressing-stands, and lockers were a model of good order and cleanliness. The lockers for some reason have not been ventilated at the back, but this is an oversight which the carpenter could speedily remedy, and no doubt this will be done now that attention has been called to the point. Much has been written upon the advantages of cross-ventilation in hospital wards. It will be remembered that on one side of each of the new wards it was not possible to continue the windows, owing to structural difficulties, although large ventilators were inserted as an alternative. Anyone criticising these wards on paper might be led to conclude that they were deficient for this reason; but the omission of the windows has brought out a useful practical point. It is found in practice that the three cots which are placed along that portion of the wall which contains no windows are most useful for the reception of severe cases, requiring a modified light and relative quietude. So the structural difficulties, which proved insurmountable in the present case, have resulted in producing a ward, which for administrative and medical reasons is probably, on the whole, more efficient than it would have been had cross-ventilation been continued throughout.

Some Appliances and Furniture.

Many of the fittings at this hospital are excellent. The new baths, with bevelled porcelain edges, present a most tempting appearance, as all baths should certainly do. The slop-sinks, too, have been constructed with some regard to the use to which they are put, and so the outlet has been made of the same diameter as a soil-pipe, without which this class of fitting is not only of no value, but may become a positive danger to the nurses should it be kept in continuous use. Why makers of such appliances should not have grasped the idea that a slop-sink requires an outlet equal in diameter to the soil-pipe, seeing the purposes to which it is put, we have never been able to understand. The fact remains, however, and where the committee is in doubt as to the knowledge and experience possessed by the architect they employ, they might usefully put the question, "What is the diameter of the outlet to the slop-sinks which you propose to fix?" At Great Ormond Street there is an excellent dressing-stand, which contains drawers, which take out, with tin linings, for each class of dressing used; and proper divisions for the more common instruments, for lotions, and other outward applications, &c. The whole is mounted on indiarubber wheels, can be readily moved about from cot to cot, and is altogether so handy, convenient, and good, that we commend it for adoption by hospital authorities generally. It is the practice to use crimson as the colour for the screens, and also for the counterpanes, and for the coverings of the ward-tables. This colour brightens the wards and relieves the more sombre colours of the glazed tiles which have been largely introduced throughout the buildings. The general appearance of the hospital at the present time from the mortuary to the oldest wards is attractive and pleasant. We can quite understand that some of the older Sisters have become so attached to the old wards that they do not care to move into the larger and more magnificent ones, which are now available for the treatment of patients. Altogether, the Hospital for Sick Children in Great Ormond Street is one which London has good reason to be proud of, and we regret to hear that from some cause the numbers of influential and representative people who used to visit this institution with commendable frequency, have of late diminished. This must be due to some reason which the committee should ascertain, with a view to the early re-establishment of the continuous interest of everybody who can in any way promote the prosperity of this noble charity.

A Few General Suggestions.

We would suggest that the committee, or that some of the lady governors should organise a Flower Fund, out of which flowers can be purchased for all the wards, especially during the winter months. It is too much the custom in hospitals to leave the supply of flowers to the nurses and sisters, who have often to supply them by purchases out of their own limited earnings, which are all too small for their own personal necessities. We have felt for a long time that this question of flowers, and the decoration of the wards at festivals and on great occasions, should be taken in hand by the responsible authorities, and that a special fund should be instituted so as to relieve the nursing staff of a heavy charge upon their limited resources, which ought never to have been allowed to devolve upon them at all. We were pleased to find that the system of training probationers at this hospital presents several excellent features. One of these is that every probationer is taught to be systematically cleanly in all respects. Each day has its own allotted work, which includes a certain amount of washing and ironing; the scrubbing of the bed-boards used as tables for the cots; the polishing of the brass-work on the cots, and the cleaning of the relatively large amount of glass which each sister has charge of. It has been said with truth and force that no man is fit to be placed over a number of other men until he has learned from personal experience what constitutes a day's work in every department of the business he has to conduct. So no woman is fit to be appointed to the office of sister, lady superintendent or matron, unless, during her period of probation, she has been taught—as Miss Nightingale wisely laid down nearly thirty years ago—to do every part of the work which will devolve upon any member of the staff who may ultimately come under her control and management. We congratulate all who are responsible for the training of nurses at this hospital on the wisdom and courage which they have displayed in the regulations now in force for probationer nurses. We have two more suggestions to make: One to the committee, that hydraulic lifts for patients should be provided in the well of the staircases, on the model of those at the General Infirmary, Sheffield. Another to the governors, or to those of the governors who take an active interest in the institution, that they should present an American organ to each of the wards, as our experience goes to show that music affords immense pleasure to children, and tends to inculcate marked cheerfulness, which, as we have often said, places them on the high road to recovery.

